# Fecal Microbiota Transplantation from APP/PS1 Mice Induces Th17-Related Inflammatory Parameters and Pathological Changes in the Gut–Brain Axis of Healthy C57BL/6J Mice

**DOI:** 10.3390/ijms27062791

**Published:** 2026-03-19

**Authors:** Dongni Lei, Chaomeng Zhou, Hao Zheng, Yu Kang, Zhiyong Yan

**Affiliations:** School of Life Science and Engineering, Southwest Jiaotong University, Chengdu 610031, China; ldn@my.swjtu.edu.cn (D.L.); 2018115692@my.swjtu.edu.cn (C.Z.); zh2384363107@my.swjtu.edu.cn (H.Z.); kangyu@my.swjtu.edu.cn (Y.K.)

**Keywords:** Th17 cells, microbiota, neuroinflammation, gut-brain axis, immune homeostasis, FMT, 16S rDNA, Alzheimer’s disease, Interleukin-17A

## Abstract

The gut–brain axis is increasingly implicated in Alzheimer’s disease (AD) pathogenesis, but the potential correlation between AD-associated gut microbiota and central inflammation remains largely unclear. This study aimed to explore their correlative link, with a focus on changes and involvement of Th17 cell-related factors in the gut–brain axis. Healthy C57BL/6J mice were pretreated with antibiotics for 1 week to deplete the indigenous gut microbiota, followed by 2 weeks of fecal microbiota transplantation (FMT) using feces from APP/PS1 AD model mice. Hematoxylin–eosin (H&E) staining, ELISA, reverse transcription-quantitative polymerase chain reaction (RT-qPCR), 16S rDNA sequencing, and correlation analysis were performed to evaluate ileal and central pathological changes, Th17 cell-related inflammatory mediators, ileal microbiota composition, and their potential correlations. The results demonstrated that AD-FMT significantly induced ileal inflammatory infiltration and central inflammation in recipient mice, which was accompanied by abnormal expression of Th17 cell-related indicators, elevated levels of Th17-associated inflammatory factors, upregulated RORγt mRNA expression, and perturbed ileal microbiota composition. Correlation analysis further suggested that specific ileal bacterial taxa were closely correlated with Th17 cell-related inflammatory factors. These findings suggest a potential correlation between AD-associated microbiota and central inflammation, possibly by regulating intestinal Th17 cell-related indicators and altering gut microbial composition. This study provides correlative evidence supporting the involvement of the gut–brain axis in AD-related pathogenesis, highlighting the link between gut microbiota, central inflammation and Th17-related factors.

## 1. Introduction

Alzheimer’s disease (AD) is a prevalent neurodegenerative disorder, primarily characterized by progressive cognitive decline [1]. However, there are still numerous limitations in the diagnosis and treatment of AD [2]. In recent years, since the concept of the gut–brain axis was introduced [3], the impact of gut microbiota on brain function has garnered considerable attention. Consequently, research on the gut microbiota in Alzheimer’s disease has increased significantly. On one hand, preclinical studies using mouse models have shown that transplanting microbiota from healthy donors to Alzheimer’s disease recipients can restore the balance and structure of the gut microbiome, thereby alleviating cognitive dysfunction in recipient animals [4]. On the other hand, evidence from both mouse models [5] and exploratory human studies [6] indicates that gut microbiota from Alzheimer’s disease donors can induce cognitive impairment [7], neuroinflammation, and related pathological phenotypes in healthy recipients. This process is predominantly influenced by the gut microbiota, with mechanisms including, but not limited to, damage to the intestinal barrier [8], activation of inflammatory pathways by lipopolysaccharides [9], reduction in beneficial short-chain fatty acids [10], elevation of trimethylamine [11], and the activation of inflammatory factors [12] entering the central nervous system.

T helper 17 cells (Th17 cells), originating primarily in the gut [13], are of significant interest due to their unique distribution and differentiation from CD4^+^ T cells. IL-6 and TGF-β are essential cytokines for Th17 cell differentiation [14], their combined induction promotes the expression of the specific transcription factor RORγt and drives the differentiation of CD4^+^ T cells into Th17 cells [15], with IL-1β [16] and IL-23 [17] also involved in regulating this process. As potent pro-inflammatory cells, Th17 cells secrete several key pro-inflammatory effector cytokines, including the core major effector molecule IL-17A [18], its homolog IL-17F, and IL-22 [19]. IL-17A and IL-22 are co-expressed in Th17 cells and coordinately mediate inflammatory responses [20]. During intestinal dysbiosis and damage, Th17 cells undergo extensive differentiation and secretion of pro-inflammatory factors, exacerbating injury and inflammation. Furthermore, the relationship between Th17 cells and the gut microbiota is particularly close, with certain bacteria directly promoting Th17 cell differentiation [21], while metabolites from the gut microbiota can indirectly influence this process [22]. In the context of the gut–brain axis, studies have identified pathogenic Th17 cells in the brain during chronic colitis, which can induce hypothalamic inflammation, suggesting the potential migration of Th17 cells from the peripheral immune system to the central nervous system [23]. In Alzheimer’s disease (AD) mouse models, peripheral Th17 cells can infiltrate the brain through the blood–brain barrier, exerting inflammatory effects such as inducing neuronal damage, clearing amyloid-beta (Aβ), and promoting tau protein phosphorylation [24]. We hypothesize that the gut microbiota from AD mice may regulate Th17 cells, leading to central nervous system damage in healthy mice.

**Figure 1:**
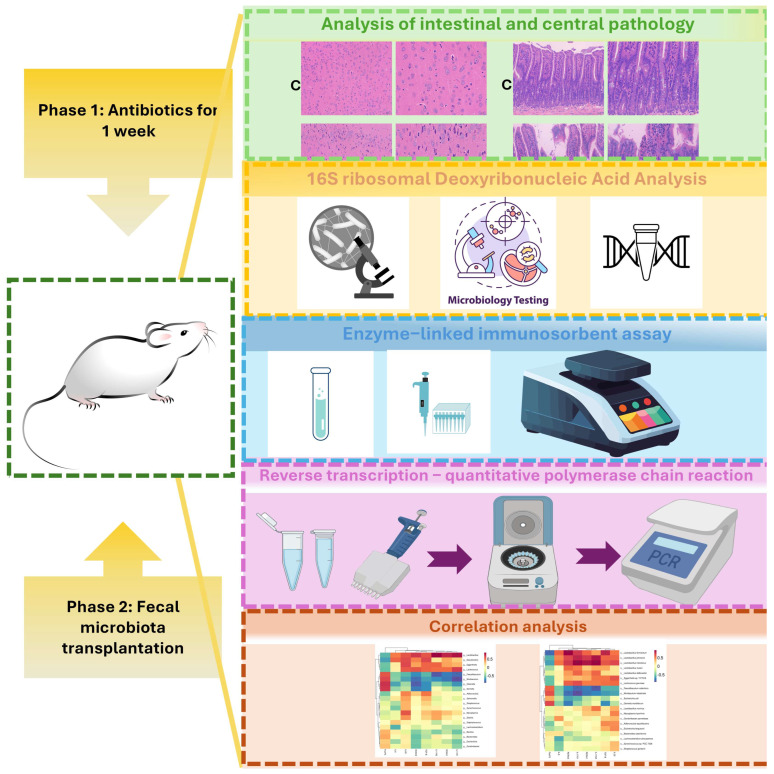
Overview of experimental research. (* *p* < 0.05, ** *p* < 0.01).

The primary objective of this descriptive and correlative study is to explore whether gut microbiota from transplanted AD model mice alone is sufficient to induce abnormal changes in Th17 cell-related factors such as IL-17 in healthy recipient mice, and further to investigate the potential correlative link between these changes and central inflammation. Although emerging evidence has linked gut dysbiosis with neuroinflammation in AD, direct in vivo evidence associating AD-associated gut microbiota with intestinal Th17 activation and corresponding central inflammatory phenotypes remains limited. This study was designed to provide descriptive and quantitative correlative evidence for this potential link. Clarifying this association may improve our understanding of the gut–brain axis in AD pathogenesis and support the identification of potential targets within the gut microbiota–Th17 cell–neuroinflammation axis. Figure 1 shows a schematic overview of the experimental design.

## 2. Results

### 2.1. The Transplantation of Microbiota from APP/PS1 Mice Induced a Degree of Intestinal Dysregulation and Central Nervous System Injury in Healthy Mice

**Figure 2:**
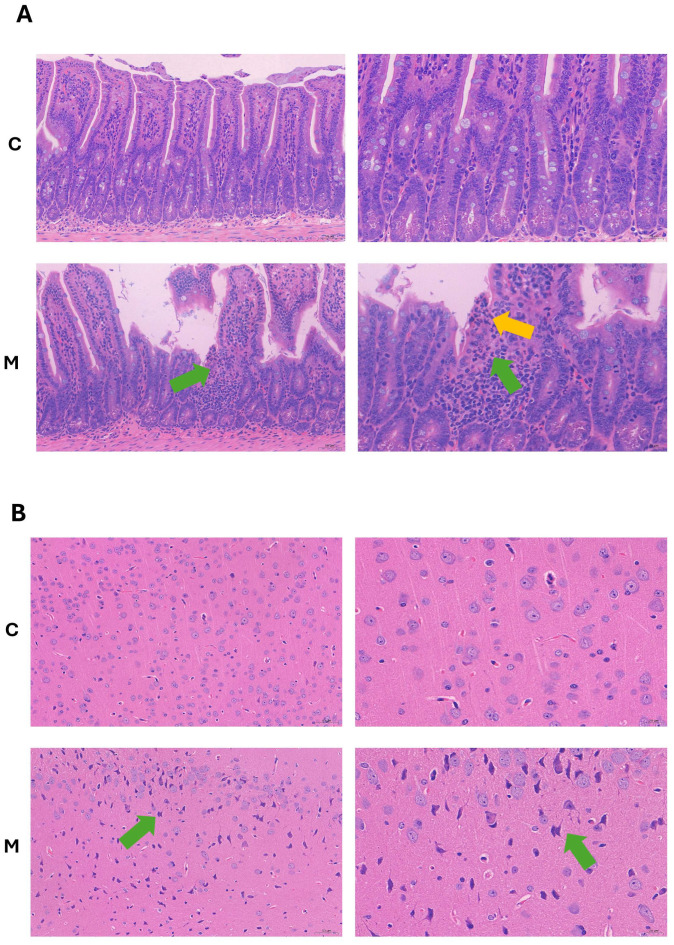
Histological staining of murine pathological sections. (**A**) H&E staining histological examination of Ileum. (**B**) H&E staining of histological examination the cerebral cortex. For the left panel of the tissue section images, the observation was conducted at ×200 magnification, with a corresponding scale bar of 50 μm; for the right panel, the magnification was set to ×400, and the scale bar was adjusted to 20 μm. *n* = 3/group, all these microscopic observations were performed using 3 biological replicates per experimental group. Abbreviations: H&E, hematoxylin and eosin.

Morphological and structural changes in the mouse ileum following fecal microbiota transplantation (FMT) were observed by light microscopy, and representative images are presented in Figure 2A. In group C, the mucosal, submucosal, muscular, and serosal layers of the small intestine tissue were relatively intact. Conversely, in group M, the mucosal layer exhibited focal necrosis, with the necrotic tissue structure appearing blurred (as indicated by the green arrow). Additionally, necrotic cells showed nuclear pyknosis, karyorrhexis, and karyolysis, accompanied by infiltration of surrounding inflammatory cells (neutrophils) (as indicated by the yellow arrow).

Histological examination of cerebral cortex slices revealed that group C maintained an intact pia mater structure and normal neuronal morphology (Figure 2B). In contrast, group M exhibited an increased number of darkly stained neurons with shrunken cell bodies and ambiguous or absent internal cellular structures. Axon-like protrusions were observed at one end of some cell bodies. These morphological features represent lesions compatible with neuronal injury. Collectively, these observations suggest that morphological alterations occurred in both the intestine and central nervous system of mice following FMT.

### 2.2. Alterations in Gut Microbiota

**Figure 3:**
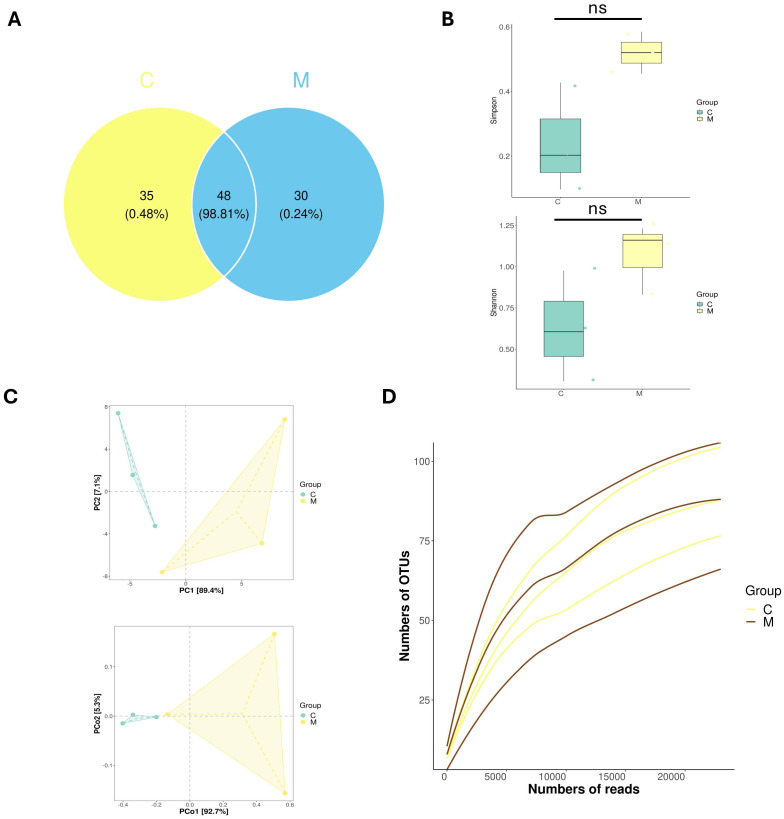
Diversity of ileal microbiota in mice. (**A**) Petal plot of community composition. (**B**) The alpha diversity (Simpson and Shannon). (**C**) The beta diversity (PERMANOVA R^2^ = 0.60, *p* = 0.10). (**D**) Rarefaction curve. (*n* = 3/group), ns, not significant, compared to the group C. Abbreviations: PCoA, Principal Co-ordinates Analysis; PC, Principal Component; OTUs, Operational Taxonomic Units. For (**A**), Unclassified/unidentified taxa were excluded.

To assess the impact of FMT on mouse ileal microbiota, we performed an analysis of the ileal microbiota composition in experimental mice. The Venn diagram (Figure 3A) visually presents the shared and unique operational taxonomic units (OTUs) across the two experimental groups. Analyses revealed 48 shared species between Group C and Group M; furthermore, Group C harbored 35 unique species, while Group M had 30 unique species. Subsequent diversity analyses revealed changes in microbial community composition across the two groups. From the α-diversity (Figure 3B), the visualizations, presented top to bottom, show the Simpson Index and Shannon Index, respectively. These plots indicate a trend toward higher microbial α-diversity in Group M than in Group C, although the difference did not reach statistical significance (*p* > 0.05). β-diversity analysis (Figure 3C) was performed to evaluate microbial community composition differences between groups. In the ordination plot, samples from Group C and Group M showed a trend of differential distribution along the coordinate axes. PERMANOVA analysis indicated that group membership explained 60% of the total variation in community composition (R^2^ = 0.60, *p* = 0.10), and no statistically significant difference in microbial community structure was observed between the two groups. Collectively, these FMT elicits alterations in the composition of the ileal microbiota in recipient mice. The rarefaction curve (Figure 3D) serves as an indirect indicator of species richness within each sample: when the curve reaches a plateau, it signifies that the sequencing depth is sufficient to cover all detectable species in the sample, ensuring the sequencing data adequately reflects the true species richness of the ileal microbiota.

**Figure 4:**
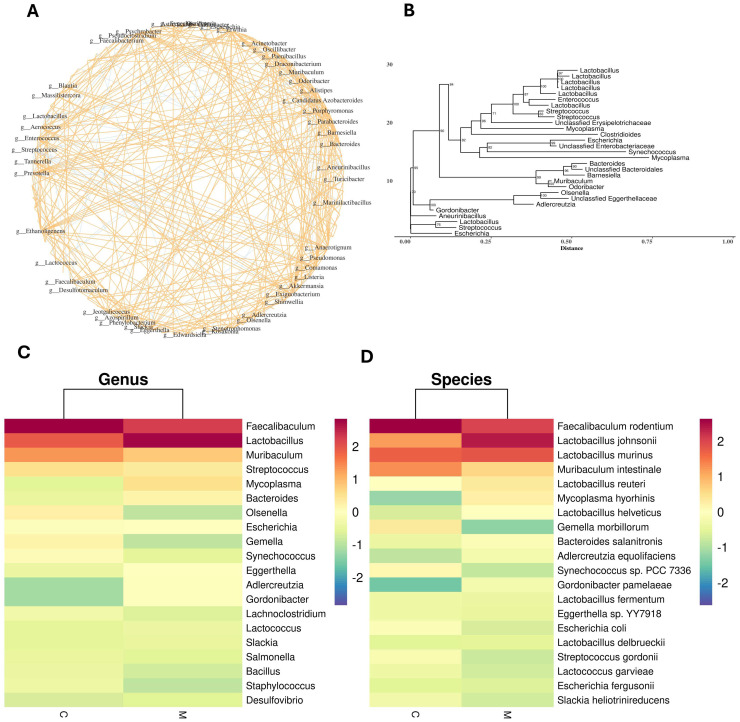
Composition of microbial communities in the ileum of mice. (**A**) Community composition network diagram. (**B**) Phylogenetic tree. (**C**) Genus-level microbial heatmaps. (**D**) Species-level microbial heatmaps. Data are expressed as mean ± SEM, *n* = 3/group.

To visually illustrate the shifts in microbial community composition across groups, firstly, at the genus level, the community composition network diagram visually presents the structural characteristics of species within the community (Figure 4A). Figure 4B presented a phylogenetic tree at the genus level, marked group-specific clustering and phylogenetic differentiation were observed between the gut microbiota of Group C and Group M. Figure 4C,D present heatmaps illustrating community composition at the genus and species levels, respectively. These heatmaps convey key information, including the diversity of species groups and their variations across samples, with abundance represented by color intensity. To facilitate clearer visualization of these variations and to prevent uneven color distribution, we standardized the rows. Combined with the figures, the characteristic clade of the group C was dominated by the *Faecalibaculum*, which formed a tightly clustered branch in the phylogenetic tree with a bootstrap support value of ≥96% and thus represented the core phylogenetic signature of the control group’s microbiota. In contrast, the group M harbored a characteristic clade dominated by the *Lactobacillus*, which constituted an independent, highly supported branch with a bootstrap support value of ≥97% in the tree. Notably, a substantial phylogenetic distance was detected between the characteristic clades of the two groups, indicating that FMT not only led to the replacement of dominant microbial genera but also induced a significant reshaping of the overall phylogenetic structure of the gut microbiota.

**Figure 5:**
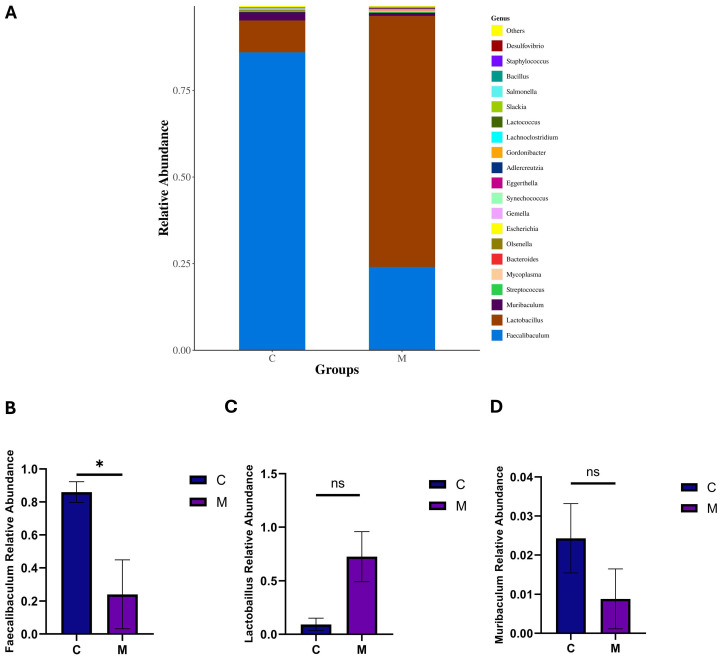
Analysis of the top three ileal flora at the genus level (**A**) Genus-level microbial abundance. (**B**) The relative abundance of *Faecalibaculum*. (**C**) The relative abundance of *Lactobacillus*. (**D**) The relative abundance of *Muribaculum*. Data are expressed as mean ± SEM, *n* = 3/group; * *p* < 0.05, ns, not significant. For (**A**), Unclassified/unidentified taxa were excluded.

To further elucidate the structural composition of the microbial communities in each group, a bar graph was generated to display the top 20 taxa (at both the genus and species taxonomic levels) among the two experimental groups. At the genus level (Figure 5A), *Faecalibaculum*, *Lactobacillus*, and *Muribaculum* were determined to be the dominant bacterial genera in the mouse ileal microbiota. Separate bar charts were generated for the top three genera and species to facilitate comparison. Relative to the group C, the relative abundance of *Faecalibaculum* was significantly reduced in the group M (*p* < 0.05) (Figure 5B). *Lactobacillus* exhibited a distinct increase in relative abundance, while *Muribaculum* showed a clear decrease, yet neither change reached statistical significance. (Figure 5C,D).

**Figure 6:**
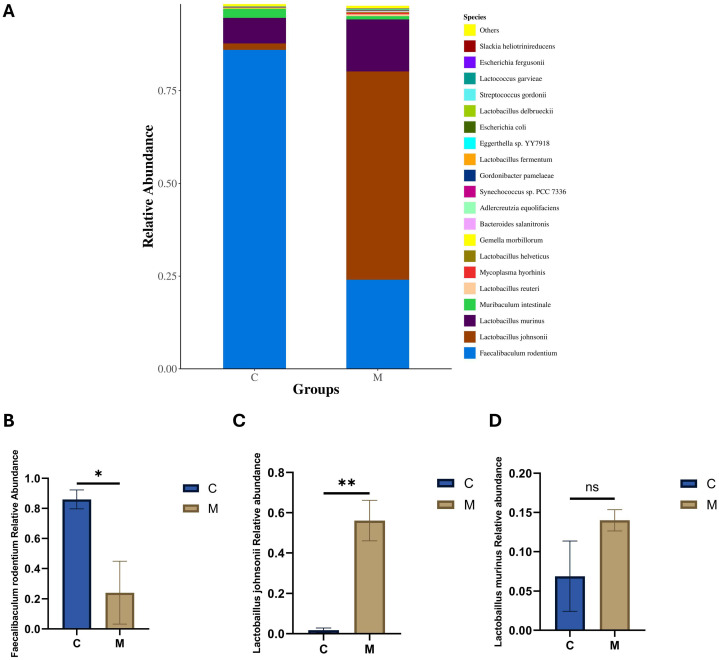
Analysis of the top three ileal flora at the species level (**A**) Species-level microbial abundance. (**B**) The relative abundance of *Faecalibaculum rodentium*. (**C**) The relative abundance of *Lactobacillus johnsonii*. (**D**) The relative abundance of *Lactobacillus murinus*. Data are expressed as mean ± SEM, n = 3/group; * *p* < 0.05, ** *p* < 0.01, ns, not significant. For (**A**), Unclassified/unidentified taxa were excluded.

Variations at the species level are more indicative of the regulatory alterations occurring in specific microbial species. The bar chart shows (Figure 6A) that the top three putative bacterial species are *Faecalibaculum rodentium*, *Lactobacillus johnsonii*, and *Lactobacillus murinus*. At the annotated species level, compared to group C, group M showed a significant reduction in putative *Faecalibaculum rodentium* (*p* < 0.05) (Figure 6B), whereas *Lactobacillus johnsonii* was significantly higher than in groups C (*p* < 0.01) (Figure 6C). *Lactobacillus murinus* showed an increase in group M; however, the difference between the two groups was not statistically significant. In summary, the results indicate that fecal microbiota transplantation altered and disrupted the microbial community in mice (Figure 6D).

### 2.3. Changes in the Levels of Th17 Cell-Related Factors

#### 2.3.1. Ileum

**Figure 7:**
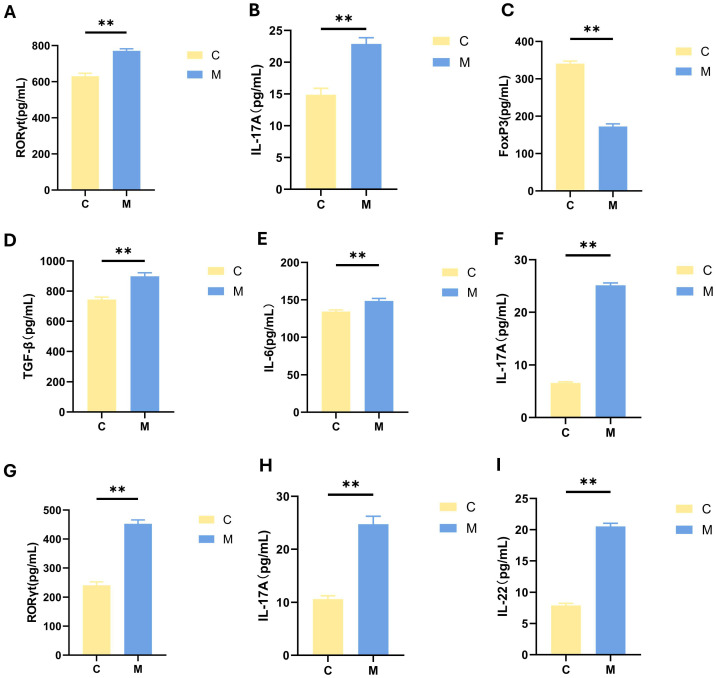
Changes in factors related to Th17 cells as measured by ELISA. (**A**) RORγt in the ileum FoxP3 in the ileum. (**B**) IL-17A in the ileum. (**C**) FoxP3 in the ileum. (**D**) TGF-β in serum. (**E**) IL-6 in serum. (**F**) IL-17A in serum. (**G**) RORγt in the hippocampus. (**H**) IL-17A in the hippocampus. (**I**) IL-22 in the hippocampus. Data are expressed as mean ± SEM, n = 9/group; ** *p* < 0.01. Abbreviations: RORγt, Retinoid-related Orphan Receptor Gamma T; FoxP3, Fork-head Box P3; IL-17A, Interleukin-17A; IL-6, Interleukin-6; TGF-β, Transforming Growth Factor-β; IL-22, Interleukin-22; ELISA, enzyme-linked immunosorbent assay.

Ileal concentrations of RORγt, FoxP3 and IL-17A were quantified across the two experimental groups via ELISA. As presented in Figure 7, the concentrations of RORγt and IL-17A in the ileum were significantly elevated in group M relative to group C. (Figure 7A,B, *p* < 0.01), whereas the concentration of FoxP3 was notably reduced (Figure 7C, *p* < 0.01). This observation reflects an alteration in intestinal immune homeostasis, characterized by an increase in the transcription factors of Th17 cells and enhanced secretion of the pro-inflammatory cytokine IL-17A, alongside a decrease in the FoxP3 expression.

#### 2.3.2. Serum

Serum concentrations of IL-6 and TGF-β (key cytokines associated with Th17 cell function) were measured in each group, together with serum IL-17A, a major pro-inflammatory factor secreted by Th17 cells. Additionally, we measured the levels of IL-17A in the serum, the inflammatory factors secreted by Th17 cells.

Relative to the healthy group C, the group M showed significantly elevated serum levels of both IL-6, TGF-β and IL-17A (Figure 7D–F, both *p* < 0.01). These results demonstrated increased inflammatory and Th17 cell differentiation-related factors in peripheral serum following FMT.

#### 2.3.3. Hippocampus

In the hippocampal tissues, the levels of IL-17A, RORγt and IL-22 in the group M exhibited a significant elevation compared to the group C (Figure 7G–I, both *p* < 0.01). These findings indicate that Th17 cell-related inflammatory alterations also occur in the hippocampus.

### 2.4. Expression of Th17 Cells and Factors

#### 2.4.1. Expression of RORγt, IL-17A and FoxP3 in the Ileum

**Figure 8:**
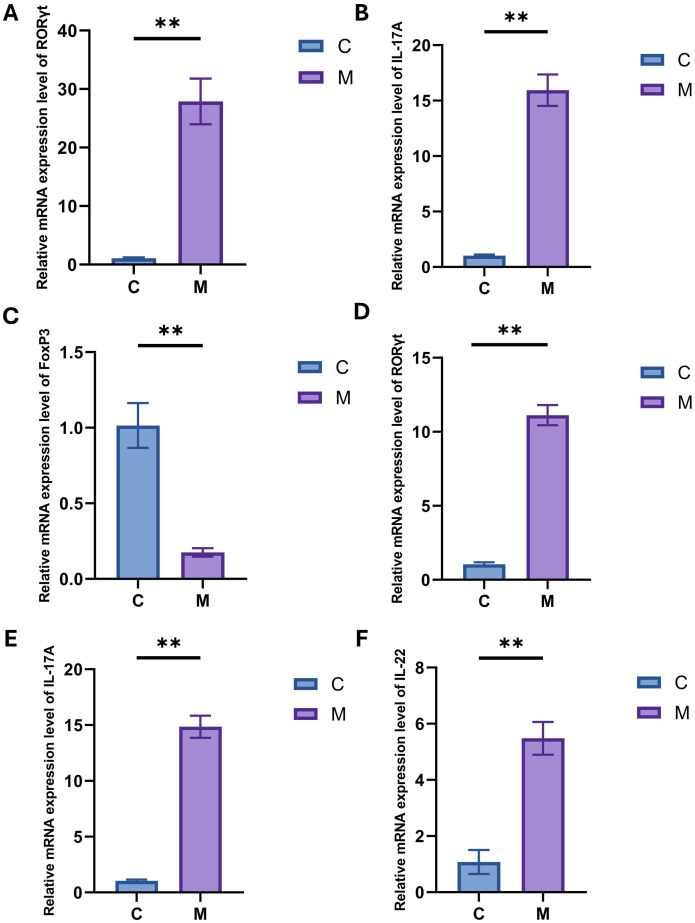
RT-qPCR analysis of Th17-related factor expression in the ileum and hippocampus. (**A**) The expression of RORγt in the ileum. (**B**) The expression of IL-17A in the ileum. (**C**) The expression of FoxP3 in the ileum. (**D**) The expression of RORγt in the hippocampus. (**E**) The expression of IL-17A in the hippocampus. (**F**) The expression of IL-22 in the hippocampus. Data are expressed as mean ± SEM, *n* = 7/group; ** *p* < 0.01. Abbreviations: RT-qPCR, Quantitative Real-Time Reverse Transcription Polymerase Chain Reaction.

To assess the association between the microbiota of model mice and intestinal and central Th17 cells, we measured the mRNA expression levels of related factors. As shown in Figure 8A–C, the expression of RORγt and IL-17A mRNA in the ileum of group M was significantly increased (*p* < 0.01), while the expression of FoxP3 was significantly decreased (*p* < 0.01). These results demonstrate upregulated mRNA expression of the Th17-related transcription factor RORγt and its effector cytokine IL-17A in the ileum of mice following FMT.

#### 2.4.2. Expression of RORγt, IL-17A and IL-22 in the Hippocampus

We measured the expression of RORγt, IL-17A, and IL-22 in the hippocampus (Figure 8D–F). The results indicated that the mRNA expression levels of these factors were significantly elevated in the M group compared to the C group (*p* < 0.01). These findings indicate an association between gut microbiota from AD model mice and increased mRNA expression of Th17-related molecules in the hippocampus.

### 2.5. Correlation Between Th17 Cell-Related Factors and Ileal Microbiota

**Figure 9:**
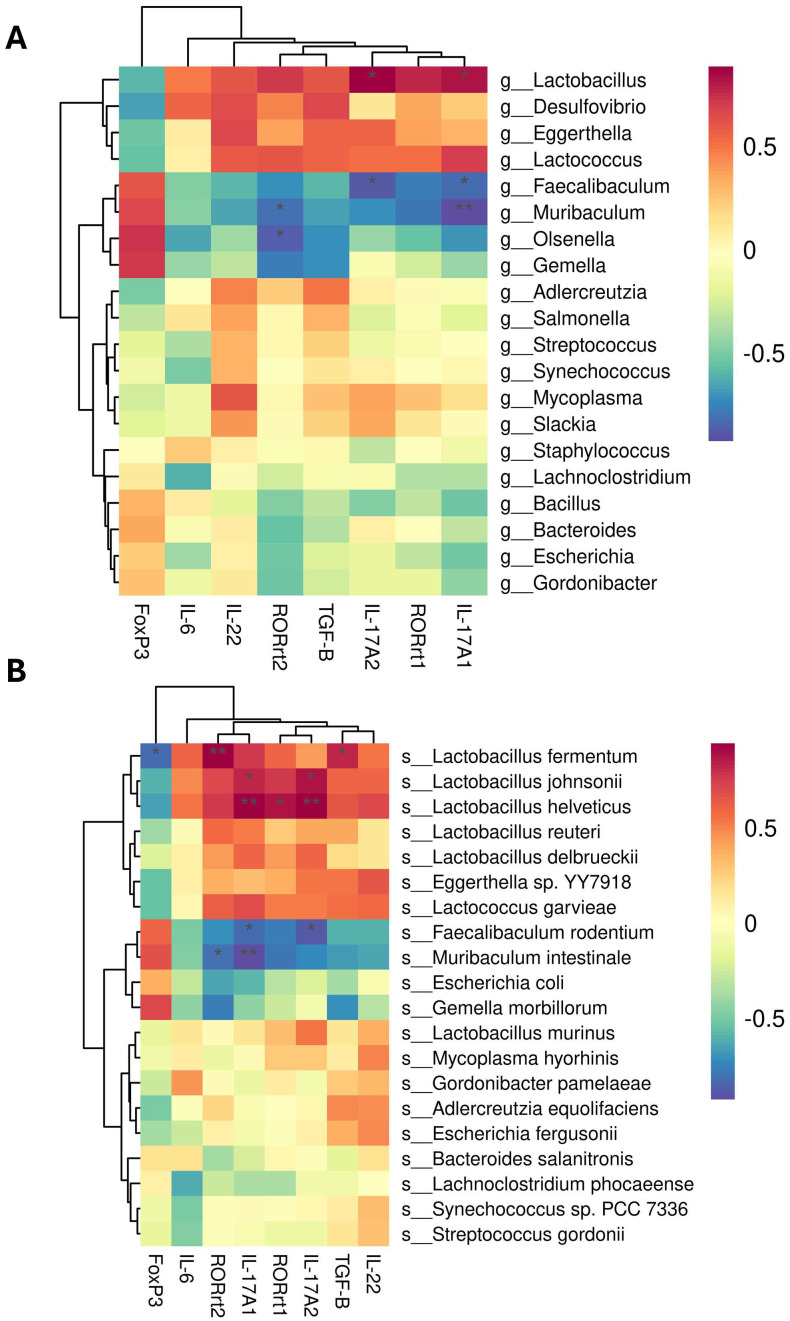
Spearman’s correlation analysis. (**A**) Genus-level correlation analysis. (**B**) Species-level correlation analysis. * *p* < 0.05, ** *p* < 0.01. Red indicates positive correlation, whereas blue indicates negative correlation. RORγt1, RORγt in the ileum. RORγt2, RORγt in the hippocampus. IL-17A1, IL-17A in the ileum. IL-17A2, IL-17A in the hippocampus.

To investigate the correlation between inflammatory factors associated with Th17 cell differentiation and ileal microbiota, spearman’s correlation analysis was utilized to investigate the top 20 most abundant microorganisms at both the genus and species taxonomic levels (Figure 9). Correlation analysis was performed using 3 randomly selected mice per group with paired microbiota sequencing and ELISA data. In this figure, for clarity, RORγt in the ileum is designated as RORγt1 and that in the hippocampus as RORγt2, IL-17A in the ileum and hippocampus is designated using the same nomenclature. At the genus level (Figure 9A), all factors with the exception of FoxP3 exhibited a positive correlation with *Lactobacillus*. However, only IL-17A (denoted as IL-17A1 and IL-17A2 in the figure) showed a statistically significant positive correlation with *Lactobacillus* (*p* < 0.05). *Faecalibaculum* was significantly negatively correlated with IL-17A (*p* < 0.05). Additionally, *Muribaculum* exhibited a highly significant negative correlation with ileal IL-17A (*p* < 0.01) and a significant negative correlation with hippocampal RORγt (*p* < 0.05). *Olsenella* also showed a significant negative correlation with hippocampal RORγt (*p* < 0.05).

At the species level, more detailed correlations were observed (Figure 9B). *Faecalibaculum rodentium* exhibited a significant negative correlation with IL-17A (*p* < 0.05). *Muribaculum intestinale* showed a highly significant negative correlation with ileal IL-17A (*p* < 0.01) and a significant negative correlation with hippocampal RORγt (*p* < 0.05). *Lactobacillus fermentum* had a significant negative correlation with FoxP3 (*p* < 0.05) and significant positive correlations with TGF-β and hippocampal RORγt (*p* < 0.05 and *p* < 0.01, respectively). *Lactobacillus johnsonii* displayed a positive correlation with IL-17A (*p* < 0.05), while *Lactobacillus helveticus* showed a positive correlation with IL-17A (*p* < 0.01) as well as a positive correlation with ileal RORγt (*p* < 0.05). These correlation results support an interplay between Th17 cell-related molecular expression and inflammatory factors.

## 3. Discussion

Fresh fecal samples were collected from APP/PS1 mice and transplanted into genetically identical healthy mice by oral gavage. It is known that the gut microbiota of AD model mice is distinct from that of healthy mice, with marked shifts in microbial diversity and function [25,26], particularly in the alteration of bacteria that produce short chain fatty acids and an increase in inflammation-associated bacteria [27,28]. Transplantation of microbiota from AD mice into healthy control mice has been reported to elicit intestinal inflammatory factors or activation of inflammatory pathways, which may be accompanied by impaired gut barrier function and tissue damage [29]. Under pathological conditions, changes in the intestinal flora can affect the central nervous system through multiple pathways [30]. Owing to dysbiosis of the donor intestinal microbiota, recipient animals can also develop pathological changes associated with this dysbiosis following FMT. For instance, using gut microbiota from AD mouse models to healthy C57BL/6 mice via oral gavage has been shown to cause neurological damage and memory impairment [31]. Cognitive function was not assessed in the present study, and the above statement is solely provided as background evidence from published literature. Consequently, we examined the cerebral cortex and ileal pathology of these mice under a microscope. Firstly, following FMT, we observed focal necrosis in the mucosal layer of the ileum, characterized by blurred necrotic tissue structure, pyknosis of necrotic cell nuclei, and infiltration of inflammatory cells. Simultaneously, HE staining of the cerebral cortex showed histopathological lesions consistent with neuronal injury in mice after FMT.

To explore the potential link between gut microbiota from APP/PS1 mice and pathological changes in the ileum and hippocampus of recipient mice, we analyzed ileal microbiota composition. Initially, PCA and PCoA plots indicated a tendency toward compositional differences in gut microbiota between group C and group M after FMT. However, given the small sample size and non-significant beta-diversity result, definitive clustering or separation between groups could not be established. By integrating analyses of microbial community composition, α/β-diversity, and phylogenetic tree construction, *Faecalibaculum* was preliminarily identified as the signature microbial genus of group C, whereas *Lactobacillus* represented the characteristic genus of group M. Further taxonomic analysis at the genus level demonstrated that *Faecalibaculum*—a core dominant genus in the ileal microbiota—exhibited the most prominent alteration, with a marked reduction in its abundance in group M. At the species level, the relative abundance of *Lactobacillus johnsonii* was significantly elevated in group M, whereas that of *Faecalibaculum rodentium* was significantly reduced in group M.

A potential association exists between microbial alterations and Th17 cell regulation. Some studies have suggested that certain bacterial species may modulate Th17 cell levels by regulating RORγt expression [32], and specific gut microbiota have been reported to affect bile acid metabolism, thereby influencing Th17 cell function and linking to inflammatory diseases [33]. In the present study, we observed a markedly decreased relative abundance of *Faecalibaculum rodentium* in group M. Importantly, according to previous reports, *Faecalibaculum rodentium* exerts a protective role in the gut; it participates in bile acid metabolism through the gut–liver axis [34] and is directly involved in secondary bile acid metabolism [35]. Moreover, *Faecalibaculum rodentium* has been shown to maintain intestinal homeostasis [36] by regulating eosinophil-related pathways, thereby exerting intestinal protective effects. In intestinal disease models, *Faecalibaculum rodentium* can also alleviate inflammation and disease severity via butyrate production [37]. In addition, cholesterol metabolites have been reported to influence RORγt expression in Th17 cells, which may further contribute to amyloidosis [38]. Based on the above literature and our current observations, we hypothesize that cholesterol metabolism may be closely associated with gut microbiota, and bile acids, as important cholesterol metabolites, can be further transformed by gut microbes, which in turn promotes Th17 cell differentiation [39]. This interpretation remains hypothetical and awaits direct experimental validation.

Notably, we observed that the relative abundances of *Lactobacillus* at the genus level and *Lactobacillus johnsonii* at the species level were both significantly increased in group M. These taxa are widely recognized to exert protective roles in the intestinal microecosystem [40], which may raise several speculative interpretations. One speculative interpretation is that the elevation of *Lactobacillus* may represent a compensatory response of the gut microbiota: the dysbiosis induced by APP/PS1-derived microbiota may trigger a compensatory proliferation of *Lactobacillus.* For example, after the reduction in certain short-chain fatty acid-producing bacteria, *Lactobacillus* may expand to help maintain intestinal barrier homeostasis [41]. However, such a response may only represent partial compensation and may not be sufficient to prevent the progression of intestinal or neural disturbances. Another plausible hypothesis is that gut dysbiosis elicits immune and inflammatory reactions, and the increased *Lactobacillus* may participate in tryptophan metabolism in response to such immune activation [42], thereby regulating Th17 cell levels. A further alternative speculation is that microbial dysbiosis may lead to nutritional competition and niche alterations, causing transient microbial overgrowth and metabolic disorders, which may accelerate inflammatory progression. In addition, interindividual differences among fecal donors and recipients, as well as temporal and environmental variations, may also contribute to this observation [43]. All the above mechanistic interpretations are speculative and not supported by direct experimental evidence in this study, and further functional and metabolomic investigations are required to verify these hypotheses.

We hypothesize that bacterial groups such as *Faecalibaculum rodentium* may be associated with the levels and expression of Th17 cells in the intestinal environment. We employed RT-qPCR and ELISA methodologies to quantify the transcription factors and secreted inflammatory cytokines of Th17 cells at two distinct levels. These metrics are instrumental in assessing the differentiation state of Th17 cells. Previously, we discussed that Th17 cells produce IL-17A and IL-22.Both IL-17A and IL-22 have the potential to compromise the blood–brain barrier, thereby exacerbating neuroinflammation [44,45]. Recent studies suggest that IL-17A may exhibit “migratory” properties [46], transferring from the intestinal tract to the lungs, thereby contributing to an inflammatory cycle. IL-6 and TGF-β are essential factors for Th17 cell differentiation; fluctuations in their serum concentrations may reflect the differentiation status of Th17 cells. Consistent with the enhanced inflammatory response mediated by intestinal Th17 cells, this finding provides serological evidence for peripheral signal changes associated with intestinal inflammation driven by Th17 cell activation. FoxP3, the transcription factor for Treg cells, typically exerts a protective, anti-inflammatory effect [47]. During pathological conditions, FoxP3 downregulation due to immune imbalance can lead to a vicious cycle of inflammation, indirectly reflecting the disease state [48,49]. Therefore, in our experiments, in addition to the aforementioned key factors, we also examined the expression and concentration of FoxP3. Following FMT, mice exhibited significant increases in the levels of key factors associated with Th17 cell secretion, including IL-17A and IL-22, within the ileum, serum, and hippocampus. Our RT-qPCR results indicated that the inflammation mediated by Th17 cell proliferation was driven by an upregulation at the transcriptional level.

To further clarify the relationship between these two variables, correlation analysis was performed. The findings were consistent with our previous microbiota analysis: significant correlations were observed for the genera *Faecalibaculum* and *Lactobacillus*, along with the bacterial species belonging to these genera. Based on the above findings, we hypothesize that the gut microbiota, particularly *Faecalibaculum rodentium*, may be associated with intestinal immune alterations and Th17 cell-related changes observed in this study. These results support a potential connection within the microbiota–gut–Th17–brain axis. This study, utilizing a fecal microbiota transplantation model, suggests a potential mediating role of the Th17 cell-associated cytokines in central inflammation linked to Alzheimer’s disease-associated gut microbiota in healthy mice. We identified this axis as a potential link for inflammatory signaling along the gut–brain axis in AD, thereby addressing a key gap in understanding how gut microbiota is associated with central inflammatory processes in this disease. Furthermore, our work highlights the gut microbiota and Th17 cell-related immune alterations as promising dual targets for future AD intervention strategies, thereby providing important theoretical guidance for the development of novel preventative and therapeutic approaches. Additionally, our findings support Th17 cells and their downstream effectors (e.g., IL-17A) as promising non-invasive biomarkers for preclinical AD early detection, providing a new strategy for developing early screening tools.

Our study has several limitations. First, direct identification and quantification of Th17 cells by flow cytometry were not performed, Western blot analysis was not conducted, and mechanistic intervention experiments were also lacking in the present study, which restrict direct phenotypic verification and mechanistic validation. Second, the sample size employed for gut microbiota analysis was relatively limited, which may affect the robustness and generalizability of the related results. Third, the inclusion of additional control groups was lacking in the current experimental design. In addition, the observed correlations between specific gut microbiota and Th17-related cytokines remain merely associative; further studies integrating metabolomics, proteomics, and other multi-omics strategies will be necessary to elucidate the underlying mechanisms and validate our preliminary findings. Future research will adopt larger sample sizes for gut microbiota profiling, incorporate additional control groups, perform flow cytometric analysis, and utilize multi-omics approaches to further verify the potential role of the gut–Th17 cell–brain axis in AD-associated neuroinflammation.

## 4. Materials and Methods

### 4.1. Animals

Eighteen healthy SPF-grade male C57BL/6J mice (6 months old, body weight 27–35 g) were provided by SPF (Beijing) Biotechnology Co., Ltd. (Beijing, China; certificate No. SCXK (Jing) 2024-0001). Age-matched APP/PS1 mice on the same genetic background were used as fecal microbiota donors, and were also obtained from SPF (Beijing) Biotechnology Co., Ltd. (gene test report No. SPF20241107-01ZJY). All recipient mice were housed under standard conditions with a standard maintenance diet (Ke’ao Xieli Feed Co., Ltd., Tianjin, China; production license SCXK (Jin) 2020-0004) and corncob bedding produced by Dachang Hui Autonomous County Chenfu Eden Bedding Processing Factory (Hebei, China) (production license SCXK (Ji) 2021-008).

### 4.2. Reagents

Ampicillin capsules (batch number HC20140024) were provided by Federal Pharmaceutical Factory Co., Ltd. (HongKong, China); metronidazole tablets (batch number H220057236) were supplied by Sichuan Kelun Pharmaceutical Co., Ltd. (Chengdu, China); gentamicin sulfate tablets (batch number H50021076) were obtained from Chongqing Dicang Yangtze River Pharmaceutical Co., Ltd. (Chongqing, China); roxithromycin dispersible tablets (batch number National Drug Approval H19980087) were provided by Harbin Pharmaceutical Group No.6 Pharmaceutical Factory(Harbin, China). These antibiotics were prepared into a broad-spectrum antibiotic mixture using sterile normal saline, at concentrations of 1 g/L ampicillin, 1 g/L metronidazole, 100 mg/L gentamicin sulfate, and 10 mg/L roxithromycin. The final concentrations were calculated according to the labeled content of each purchased drug. The following enzyme-linked immunosorbent assay (ELISA) kits were utilized in this study: IL-17A ELISA kit (MM-0759M1), RORγt ELISA kit (MM-46372M1), IL-6 ELISA kit (MM-46372), TGF-β ELISA kit (MM-0689M1), IL-22 ELISA kit (MM-0892M1) and FoxP3 ELISA Kit (MM-44741M1). All kits were sourced from Jiangsu Meimian Industrial Co., Ltd. (Yancheng, China). The RT-qPCR kit utilized in this study was procured from Accuracy Biology(Changsha, China). The details of the kits were as follows: SteadyPure Quick RNA Extraction Kit (A7A3036), Evo M-MLV RT Mix Kit with gDNA clean for qPCR Ver.2 (A7A1199), SYBR Green Premix Pro Taq HS qPCR Kit (A7A2333).

### 4.3. Animal Grouping and Treatment

**Figure 10:**
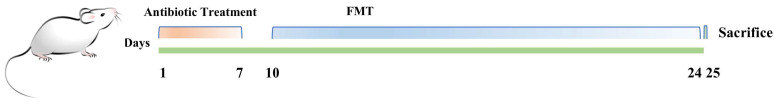
Experimental timeline. Abbreviations: FMT; fecal microbiota transplantation.

C57BL/6J mice in this study were randomly divided into two groups, with 9 mice per group and cages separated by group; the groups specifically included the control group and model group. Their respective abbreviations used throughout the study were C (control) and M (model). Their respective abbreviations used throughout the study were C (control), M (model). Prior to the formal experiment, all groups were administered the antibiotic mixture via gavage at a dose of 200 μL per mouse per day for seven consecutive days. Mice in the model group were gavaged with fresh fecal suspension from APP/PS1 mice for two consecutive weeks, while those in the control group received normal saline by gavage. During the FMT phase, the gavage volume was determined based on body weight using the formula 100 μL per 10 g of body weight (equivalent to 10 μL/g). The timeline of experimental procedures for this study is illustrated in Figure 10.

### 4.4. Fecal Microbiota Transplantation

Feces were collected from APP/PS1 mice. Fresh feces of the mice were collected. The collected feces were stored at −80 °C. For use, they were first thawed, then 0.9% normal saline was added, and the mixture was vibrated and mixed on a vortex mixer for 5 min. First, large insoluble particles in the fecal homogenate were pelleted and discarded via centrifugation at 500× *g* using a low-speed centrifuge; the resulting supernatant was then collected to prepare a fecal microbiota suspension with a final concentration of 50 mg/mL for subsequent FMT.

### 4.5. Histopathological Section Staining

Mouse hemispheres and ileum were fixed in 10% paraformaldehyde. Post-fixation, the tissues underwent dehydration, embedding, and sectioning using an automated tissue processor. The sections were subsequently deparaffinized and rehydrated in water, followed by hematoxylin staining for 5–10 min. The sections were rinsed with running tap water until colorless, differentiated with acid alcohol solution for approximately 3 s, and then washed with tap water. A weak alkaline solution was used for bluing, followed by a tap water rinse and staining with eosin for 3 min. In the final step of tissue section preparation, the sections were first dehydrated using a gradient ethanol series, then cleared with xylene (a standard histological clearing agent), and finally mounted in neutral balsam. All processed sections were observed under a light microscope at two different magnifications (×200 and ×400) to assess histopathological changes in target tissues. Detailed experimental and equipment information is provided in Supplementary Materials Table S1.

### 4.6. ELISA of Th17-Related Factors

Upon experimental completion, mice were euthanized, and serum, hippocampal tissues, and ileal tissues were harvested from each group. According to the ELISA kit instructions, the contents of Th17 differentiation factors IL-6, IL-17A and TGF-β in mouse serum, the contents of transcription factors RORγt, IL-17A and FoxP3 in mouse ileum, and the contents of IL-22, RORγt and IL-17A in mouse hippocampus were determined. The content of each factor was calculated based on the obtained standard curve.

### 4.7. RT-qPCR of the Ileum and Hippocampus

Fresh ileum and hippocampus tissues were collected and weighed following the kit instructions. Total RNA was extracted from these tissues, and samples were homogenized in lysis buffer. After purification to eliminate genomic DNA contamination, complementary DNA (cDNA) was synthesized via reverse transcription. For RT-qPCR analysis, amplification reactions were prepared using primers, cDNA templates, and RNase-free water according to the manufacturer’s protocol, and detected on a real-time quantitative PCR system. The thermal cycling conditions were set as follows: 95 °C for 30 s, followed by 40 cycles of 95 °C for 5 s and 60 °C for 30 s. The mRNA expression levels of IL-22, RORγt, and IL-17A in the hippocampus, and IL-17A, FoxP3, and RORγt in the ileum were quantified. Seven biological replicates (*n* = 7) were used in each group, and three technical replicates were conducted for each biological sample; the mean cycle threshold (Ct) values obtained from technical replicates were used for subsequent data analysis. All gene expression results were normalized to the stably expressed internal reference gene. The primer sequences and other detailed experimental information are provided in Supplementary Materials Table S2.

### 4.8. 16S rDNA Amplicon Sequencing of Intestinal Microbiota

Following euthanasia of the mice under sterile conditions, ileal contents were extracted and stored in sterile cryotubes at −80 °C for future analysis. Three samples were randomly selected from each group. The primary procedures involved the extraction of genomic DNA using a specific DNA extraction kit, followed by electrophoresis for DNA detection. For 16S rRNA gene sequencing analysis, specific primers labeled with 16S universal full-length barcodes were utilized to amplify the target variable regions of the sample DNA. The subsequent experimental workflow included sample DNA quantification, high-throughput sequencing library construction, and bioinformatic analysis of sequencing data. This entire set of 16S rRNA gene-related experiments—encompassing amplification, library preparation, and data processing—were outsourced to Chengdu Rhonin Biosciences Co., Ltd. (Chengdu, China). Detailed information on the bioinformatics analysis pipeline, sequencing platform, and taxonomic database is provided in Supplementary Materials Table S3.

### 4.9. Statistical Analyses

All experimental data were presented as mean ± SEM. Statistical analysis was performed using IBM SPSS Statistics (version 27.0.1). Independent samples t-test was used to compare differences between the two groups, after confirming that the data conformed to normal distribution and homogeneity of variance, and FDR (Benjamini–Hochberg) correction was applied to control the false-positive rate. For gut microbiota analysis, PERMANOVA was performed to evaluate significant differences in microbial community structure between groups. For Spearman correlation analyses, *p*-values were adjusted using FDR correction, and an FDR-adjusted *p* < 0.05 was considered statistically significant. For all other comparisons, a *p*-value < 0.05 was defined as statistically significant. Experimental results were visualized using bar charts and other graphical representations constructed with GraphPad Prism (v.9). Image analysis for tissue sections was carried out using Image-Pro Plus (v.6.0) software.

## Data Availability

The raw data supporting the conclusions of this article will be made available by the authors on request.

## Supplementary Materials

The following supporting information can be downloaded at: https://www.mdpi.com/article/10.3390/ijms27062791/s1.

## Abbreviations

AD	Alzheimer’s disease
FMT	Fecal microbiota transplantation
Th17 cell	T helper 17 cell
Treg cell	Regulatory T cell
H&E	Hematoxylin and eosin
IL-6	Interleukin-6
TGF-β	Transforming Growth Factor-β
ELISA	Enzyme-linked immunosorbent assay
RORγt	Retinoid-related Orphan Receptor Gamma T
FoxP3	Forkhead Box P3
IL-17A	Interleukin-17A
IL-22	Interleukin-22
IL-23	Interleukin-23
RT-qPCR	Real-Time Reverse Transcription Polymerase Chain Reaction

## References

[1] Scheltens P., Blennow K., Breteler M.M., de Strooper B., Frisoni G.B., Salloway S., Van der Flier W.M. (2016). Alzheimer’s disease. Lancet.

[2] Malek J., Levchenko A., Robinson J.O., Fong J., Lin C.R., Jackson G.R., Blumenthal-Barby J., Shulman J.M., McGuire A.L. (2025). Dilemmas in diagnosing Alzheimer’s disease: The peril and promise of self-fulfilling prophecies. J. Alzheimer’s Dis..

[3] Góralczyk-Bińkowska A., Szmajda-Krygier D., Kozłowska E. (2022). The Microbiota-Gut-Brain Axis in Psychiatric Disorders. Int. J. Mol. Sci..

[4] Li X., Ding Q., Wan X., Wu Q., Ye S., Lou Y. (2025). Fecal microbiota transplantation attenuates Alzheimer’s disease symptoms in APP/PS1 transgenic mice via inhibition of the TLR4-MyD88-NF-κB signaling pathway-mediated inflammation. Behav. Brain Funct..

[5] Wang F., Gu Y., Xu C., Du K., Zhao C., Zhao Y., Liu X. (2022). Transplantation of fecal microbiota from APP/PS1 mice and Alz-heimer’s disease patients enhanced endoplasmic reticulum stress in the cerebral cortex of wild-type mice. Front. Aging Neurosci..

[6] Zhang Y., Shen Y., Liufu N., Liu L., Li W., Shi Z., Zheng H., Mei X., Chen C.Y., Jiang Z. (2023). Transmission of Alzheimer’s disease-associated microbiota dysbiosis and its impact on cognitive function: Evidence from mice and patients. Mol. Psychiatry.

[7] Upadhyay P., Kumar S., Tyagi A., Tyagi A.R., Barbhuyan T., Gupta S. (2025). Gut Microbiome rewiring via fecal transplants: Uncovering therapeutic avenues in Alzheimer’s disease models. BMC Neurosci..

[8] Zhou C., Feng X., Liu H., Cai T., Li Y., Fan H. (2025). Bidirectional modulation of Alzheimer’s disease via gut microbiota: Rescue by fecal transplantation from healthy donors and aggravation by colitis-associated dysbiosis. Front. Neurosci..

[9] Soliman M.L., Ohm J.E., Rosenberger T.A. (2013). Acetate reduces PGE2 release and modulates phospholipase and cyclooxygenase levels in neuroglia stimulated with lipopolysaccharide. Lipids.

[10] Qian X.H., Xie R.Y., Liu X.L., Chen S.D., Tang H.D. (2022). Mechanisms of Short-Chain Fatty Acids Derived from Gut Microbiota in Alzheimer’s Disease. Aging Dis..

[11] Vogt N.M., Romano K.A., Darst B.F., Engelman C.D., Johnson S.C., Carlsson C.M., Asthana S., Blennow K., Zetterberg H., Bendlin B.B. (2018). The gut microbiota-derived metabolite trimethylamine N-oxide is elevated in Alzheimer’s disease. Alzheimer’s Res. Ther..

[12] Agirman G., Yu K.B., Hsiao E.Y. (2021). Signaling inflammation across the gut-brain axis. Science.

[13] Korn T., Bettelli E., Oukka M., Kuchroo V.K. (2009). IL-17 and Th17 Cells. Annu. Rev. Immunol..

[14] Harbour S.N., DiToro D.F., Witte S.J., Zindl C.L., Gao M., Schoeb T.R., Jones G.W., Jones S.A., Hatton R.D., Weaver C.T. (2020). T(H)17 cells require ongoing classic IL-6 receptor signaling to retain transcriptional and functional identity. Sci. Immunol..

[15] Ivanov I.I., McKenzie B.S., Zhou L., Tadokoro C.E., Lepelley A., Lafaille J.J., Cua D.J., Littman D.R. (2006). The orphan nuclear receptor RORgammat directs the differentiation program of proinflammatory IL-17+ T helper cells. Cell.

[16] Coccia M., Harrison O.J., Schiering C., Asquith M.J., Becher B., Powrie F., Maloy K.J. (2012). IL-1β mediates chronic intestinal inflammation by promoting the accumulation of IL-17A secreting innate lymphoid cells and CD4(+) Th17 cells. J. Exp. Med..

[17] Volpe E., Servant N., Zollinger R., Bogiatzi S.I., Hupé P., Barillot E., Soumelis V. (2008). A critical function for transforming growth factor-beta, interleukin 23 and proinflammatory cytokines in driving and modulating human T(H)-17 responses. Nat. Immunol..

[18] Schulz S.M., Köhler G., Holscher C., Iwakura Y., Alber G. (2008). IL-17A is produced by Th17, gammadelta T cells and other CD4- lymphocytes during infection with Salmonella enterica serovar Enteritidis and has a mild effect in bacterial clearance. Int. Immunol..

[19] Nembrini C., Marsland B.J., Kopf M. (2009). IL-17-producing T cells in lung immunity and inflammation. J. Allergy Clin. Immunol..

[20] Ouyang W., Kolls J.K., Zheng Y. (2008). The biological functions of T helper 17 cell effector cytokines in inflammation. Immunity.

[21] Atarashi K., Tanoue T., Ando M., Kamada N., Nagano Y., Narushima S., Suda W., Imaoka A., Setoyama H., Nagamori T. (2015). Th17 Cell Induction by Adhesion of Microbes to Intestinal Epithelial Cells. Cell.

[22] Duan J., Matute J.D., Unger L.W., Hanley T., Schnell A., Lin X., Krupka N., Griebel P., Lambden C., Sit B. (2023). Endoplasmic reticulum stress in the intestinal epithelium initiates purine metabolite synthesis and promotes Th17 cell differentiation in the gut. Immunity.

[23] Mickael M.E., Bhaumik S., Chakraborti A., Umfress A.A., van Groen T., Macaluso M., Totenhagen J., Sorace A.G., Bibb J.A., Standaert D.G. (2022). RORγt-Expressing Pathogenic CD4(+) T Cells Cause Brain Inflammation during Chronic Colitis. J. Immunol..

[24] Zhang J., Ke K.F., Liu Z., Qiu Y.H., Peng Y.P. (2013). Th17 cell-mediated neuroinflammation is involved in neurodegeneration of aβ1-42-induced Alzheimer’s disease model rats. PLoS ONE.

[25] Qian X., Hai W., Chen S., Zhang M., Jiang X., Tang H. (2023). Multi-omics data reveals aberrant gut microbiota-host glycerophospholipid metabolism in association with neuroinflammation in APP/PS1 mice. Gut Microbes.

[26] Shen L., Liu L., Ji H.F. (2017). Alzheimer’s Disease Histological and Behavioral Manifestations in Transgenic Mice Correlate with Specific Gut Microbiome State. J. Alzheimer’s Dis..

[27] Wang Z., Wang C., Yuan B., Liu L., Zhang H., Zhu M., Chai H., Peng J., Huang Y., Zhou S. (2025). Akkermansia muciniphila and its metabolite propionic acid maintains neuronal mitochondrial division and autophagy homeostasis during Alzheimer’s disease pathologic process via GPR41 and GPR43. Microbiome.

[28] Zhang S., Wei D., Lv S., Wang L., An H., Shao W., Wang Y., Huang Y., Peng D., Zhang Z. (2022). Scutellarin Modulates the Microbiota-Gut-Brain Axis and Improves Cognitive Impairment in APP/PS1 Mice. J. Alzheimer’s Dis..

[29] Sun J., Zhang Y., Kong Y., Ye T., Yu Q., Kumaran Satyanarayanan S., Su K.P., Liu J. (2022). Microbiota-derived metabolite Indoles induced aryl hydrocarbon receptor activation and inhibited neuroinflammation in APP/PS1 mice. Brain Behav. Immun..

[30] Sorboni S.G., Moghaddam H.S., Jafarzadeh-Esfehani R., Soleimanpour S. (2022). A Comprehensive Review on the Role of the Gut Microbiome in Human Neurological Disorders. Clin. Microbiol. Rev..

[31] Kim N., Jeon S.H., Ju I.G., Gee M.S., Do J., Oh M.S., Lee J.K. (2021). Transplantation of gut microbiota derived from Alzheimer’s disease mouse model impairs memory function and neurogenesis in C57BL/6 mice. Brain Behav. Immun..

[32] Alexander M., Ang Q.Y., Nayak R.R., Bustion A.E., Sandy M., Zhang B., Upadhyay V., Pollard K.S., Lynch S.V., Turnbaugh P.J. (2022). Human gut bacterial metabolism drives Th17 activation and colitis. Cell Host Microbe.

[33] Paik D., Yao L., Zhang Y., Bae S., D’Agostino G.D., Zhang M., Kim E., Franzosa E.A., Avila-Pacheco J., Bisanz J.E. (2022). Human gut bacteria produce Τ(H)17-modulating bile acid metabolites. Nature.

[34] Xu H., Fang F., Wu K., Song J., Li Y., Lu X., Liu J., Zhou L., Yu W., Yu F. (2023). Gut microbiota-bile acid crosstalk regulates murine lipid metabolism via the intestinal FXR-FGF19 axis in diet-induced humanized dyslipidemia. Microbiome.

[35] Morissette A., de Wouters d’Oplinter A., Andre D.M., Lavoie M., Marcotte B., Varin T.V., Trottier J., Pilon G., Pelletier M., Cani P.D. (2024). Rebaudioside D decreases adiposity and hepatic lipid accumulation in a mouse model of obesity. Sci. Rep..

[36] Cao Y.G., Bae S., Villarreal J., Moy M., Chun E., Michaud M., Lang J.K., Glickman J.N., Lobel L., Garrett W.S. (2022). Faecalibaculum rodentium remodels retinoic acid signaling to govern eosinophil-dependent intestinal epithelial homeostasis. Cell Host Microbe.

[37] Song Z., Qiao Z., Liu J., Han L., Chen X., Wang Y. (2025). Sea buckthorn berries alleviate ulcerative colitis via regulating gut Faecalibaculum rodentium-mediated butyrate biosynthesis. Phytomedicine.

[38] Wang T., Feng W., Ju M., Yu H., Guo Z., Sun X., Yang K., Liu M., Xiao R. (2023). 27-hydroxycholesterol causes cognitive deficits by disturbing Th17/Treg balance and the related immune responses in mild cognitive impairment patients and C57BL/6J mice. J. Neuroinflammation.

[39] Li D., Zhou J., Wang L., Gong Z., Le H., Huang Y., Xu C., Tian C., Cai W., Wu J. (2023). Gut microbial metabolite deoxycholic acid facilitates Th17 differentiation through modulating cholesterol biosynthesis and participates in high-fat diet-associated colonic inflammation. Cell Biosci..

[40] Liu H.Y., Li S., Ogamune K.J., Yuan P., Shi X., Ennab W., Ahmed A.A., Kim I.H., Hu P., Cai D. (2025). Probiotic Lactobacillus johnsonii Reduces Intestinal Inflammation and Rebalances Splenic Treg/Th17 Responses in Dextran Sulfate Sodium-Induced Colitis. Antioxidants.

[41] Shi H., Newton D.P., Nguyen T.H., Estrela S., Sanchez J., Tu M., Ho P.Y., Zeng Q., DeFelice B.C., Sonnenburg J.L. (2025). Nutrient competition predicts gut microbiome restructuring under drug perturbations. Cell.

[42] Zhu Y., Tao X., Yan T., Cao S., Jiang P., Zhang Z., Li L., Wu Q. (2024). Lactobacillus murinus alleviated lung inflammation induced by PAHs in mice. Ecotoxicol. Environ. Saf..

[43] Bäuerl C., Collado M.C., Diaz Cuevas A., Viña J., Pérez Martínez G. (2018). Shifts in gut microbiota composition in an APP/PSS1 transgenic mouse model of Alzheimer’s disease during lifespan. Lett. Appl. Microbiol..

[44] Gautam A.S., Pandey S.K., Balki S., Panda E.S., Singh R.K. (2025). IL-17 A Exacerbated Neuroinflammatory and Neurodegenerative Biomarkers in Intranasal Amyloid-Beta Model of Alzheimer’s Disease. J. Neuroimmune Pharmacol..

[45] Lee D., Jo H., Go C., Jang Y., Chu N., Bae S., Kang D., Kim Y., Kang J.S. (2022). The Roles of IL-22 and Its Receptor in the Regula-tion of Inflammatory Responses in the Brain. Int. J. Mol. Sci..

[46] Xie B., Wang M., Zhang X., Zhang Y., Qi H., Liu H., Wu Y., Wen X., Chen X., Han M. (2024). Gut-derived memory γδ T17 cells exacerbate sepsis-induced acute lung injury in mice. Nat. Commun..

[47] Dominguez-Villar M., Hafler D.A. (2018). Regulatory T cells in autoimmune disease. Nat. Immunol..

[48] Wang H., Hu D., Cheng Y., Gao Q., Liu K., Mani N.L., Tang A.Y., Iyer R., Gao B., Sun L. (2025). Succinate drives gut inflammation by promoting FOXP3 degradation through a molecular switch. Nat. Immunol..

[49] Boehm F., Martin M., Kesselring R., Schiechl G., Geissler E.K., Schlitt H.J., Fichtner-Feigl S. (2012). Deletion of Foxp3+ regulatory T cells in genetically targeted mice supports development of intestinal inflammation. BMC Gastroenterol..

