# Supplementary material for: Fecal Microbiota Transplantation from APP/PS1 Mice Induces Th17-Related Inflammatory Parameters and Pathological Changes in the Gut–Brain Axis of Healthy C57BL/6J Mice
Source: Int J Mol Sci. 2026 Mar 19;27(6):2791. doi: 10.3390/ijms27062791 (PMC13027225; doi:10.3390/ijms27062791)
Supplement: Supplementary file 1 [file ijms-27-02791-s001.zip › Supplementary materials.pdf]

Supplementary materials

Table S1: Histopathological Section Staining

Procedure	Item	Information
Exact detection site		Brain (cortical and diencephalic regions); Ileum (mucosa, submucosa, muscularis externa, serosa)
Tissue sectioning	Rotary microtome	Leica-2016; Leica Microsystems, Germany Core sectioning equipment; section thickness 2–3 μm (paraffin continuous sections)
Tissue dehydration	Automatic dehydrator	JT-12S; Wuhan Junjie Electronic Co., Ltd., China; Core dehydration equipment
Tissue embedding	Tissue embedding machine	BMJ-A; Changzhou Suburb Zhongwei Electronic Instrument Factory, China; Core embedding equipment
Section processing	Pathological tissue floating and baking instrument	PHY-III; Changzhou Zhongwei Electronic Instrument Co., Ltd., China; Core section treatment equipment
Image acquisition	Digital slide scanner	Pannoramic 250; 3DHISTECH, Hungary; Core imaging equipment

Note:

Histological scoring system (5-grade scale): 0 (–) = Normal, no pathological changes; 1 (+) = Slight, minimal morphological changes within the normal range; 2 (++) = Mild, easily identifiable lesions with limited severity (11%–20% of the detected tissue involved); 3 (+++) = Moderate, prominent lesions with progressive tendency and mild organ dysfunction (21%–40% of the detected tissue involved); 4 (++++) = Severe, severe lesions with obvious organ dysfunction (41%–100% of the detected tissue involved). **No moderate (+++) or severe (++++) lesions were observed in this experiment**.

Score	Lesion Severity	Grading Definition
**0 (–)**	**Normal range**	**Under the experimental conditions, minor alterations may occur considering factors including age, sex and strain, which would otherwise be regarded as deviations from the normal state**
**1 (+)**	**Slight**	**Observed changes are almost within the normal range (i.e., minimal morphological alterations)**

Score	Lesion Severity	Grading Definition
2 (++)	Mild	Lesions are easily identifiable with limited severity and may not cause any functional impairment; the involved tissue accounts for 11%–20% of the examined tissue
3 (+++)	Moderate	Lesions are prominent with a high tendency of progression, which may induce limited tissue or organ dysfunction; the involved tissue accounts for 21%–40% of the examined tissue
4 (++++)	Severe	Lesions are severe with the formation of complete pathological changes, which are expected to cause obvious tissue or organ dysfunction; the involved tissue accounts for 41%–100% of the examined tissue

Table S2: **Information for RT-qPCR**

Equipment	Model	Information
Micro-volume Spectrophotometer	NanoDrop 2000	Thermo Fisher Scientific, USA
Gene Amplifier	TC-90/G/H(b)C	Hangzhou Bioer Technology Co., Ltd., China
Real-time Fluorescent Quantitative PCR Instrument	QuantStudio™ 1 Plus	Thermo Fisher Scientific, USA

Reagents	Volume (20 μL total)	Final concentration
2×SYBR Green *Pro Taq* HS Premix	10μL	1×
Template	-	≤100ng
Prime R	0.4μL	0.2μ M
Prime F	0.4μL	0.2μ M
RNase free water	-	Up to 20μL

Primers for target genes and reference genes used in RT-qPCR

Primer	sequence(5' to 3')
RORγt-F	CCGCTGAGAGGGCTTCAC
RORγt-R	TGCAGGAGTAGGCCACATTACA
IL-17A-F	CTCCAGAAGGCCCTCAGACTAC
IL-17A-R	AGCTTTCCCTCCGCATTGACACAG

Foxp3-F	AGAGAAGACAGACCCATGCTG
Foxp3-R	CCAAGTCTCGTCTGAAGGCA
IL-22-F	GACAGGTTCCAGCCCTACAT
IL-22-R	ATCGCCTTGATCTCTCCACT
Actin-F	CACTGTCGAGTCGCGTCC
Actin-R	CGCAGCGATATCGTCATCCA

Table S3: Information for 16S rDNA

Sequencing Platform and Core Reagents/Instruments

Category	Item Name	Model/Catalog No.	Manufacturer/Supplier
Sequencing Platform	Nanopore GridION Sequencer	MinION Flow Cell (R9.4)	Oxford Nanopore Technologies
DNA Extraction Kit	BIOMICS DNA Microprep Kit	D4301	Zymo Research
Gel Recovery Kit	Zymoclean Gel Recovery Kit	D4008	Zymo Research
PCR Enzyme	MegaFi Fidelity DNA Polymerase	G896	Applied Biological Materials Inc.
PCR Instrument	PCR System	9700	Applied Biosystems®
Nucleic Acid Quantifier	Tecan F200	-	Tecan Group Ltd.
End Repair Kit	NEBNext FFPE Repair Mix	M6630	New England Biolabs (NEB)
End Repair/dA-tailing Module	NEBNext Ultra II End repair/dA-tailing Module	E7546	NEB
Barcoding Kit	Nanopore 16S Barcoding Kit	SQK-RAB204	Oxford Nanopore Technologies
Ligation Sequencing Kit	Nanopore Ligation Sequencing Kit	SQK-LSK109	Oxford Nanopore Technologies
Flow Cell Priming Kit	Nanopore Flow Cell Priming Kit	EXP-FLP002	Oxford Nanopore Technologies

Experimental Workflow of 16S rRNA Gene Amplicon Sequencing

Step	Experimental Procedure	Key Experimental Details
1	Sample gDNA Purification	Genomic DNA extracted with Zymo Research D4301 kit; DNA integrity detected by 0.8% agarose gel electrophoresis; nucleic acid quantified via Tecan F200 (PicoGreen dye method)
2	Full-length 16S rDNA PCR Amplification	Primers: 8F (5'-AGAGTTTGATCATGGCTCAG-3') and 1492R (5'-CGGTTACCTTGTTACGACTT-3'); PCR enzyme: MegaFi Fidelity DNA Polymerase (G896); 25 μL PCR system (5×MegaFi Buffer 5 μL, 10 mM dNTPs 0.5 μL, 8F/1492R 1 μL each, polymerase 0.5 μL, template DNA (10 ng/μL) 2 μL, H_2O 15 μL); PCR program: 98 ℃ pre-denaturation 30 s (1 cycle); 98 ℃ denaturation 5 s, 54 ℃ annealing 15 s, 72 ℃ extension 45 s (25–30 cycles); 72 ℃ final extension 2 min (1 cycle); 4 ℃ hold; triplicate amplifications per sample, equal mixing of linear-phase products
3	PCR Product Validation, Purification & Quantification	Target fragment detected by 1% agarose gel electrophoresis; gel extraction with Zymo Research D4008 kit; quantification via Tecan F200; equimolar pooling of qualified products
4	Library Construction	End repair with NEB M6630 and E7546; library preparation with Nanopore SQK-RAB204, SQK-LSK109 and EXP-FLP002 kits
5	High-throughput Sequencing	Sequenced on Nanopore GridION sequencer with MinION Flow Cell (R9.4)

Bioinformatics Analysis Workflow of 16S rRNA Gene Sequencing Data

Step	Analysis Item	Core Software/Database & Key Parameters
1	Basecalling	Guppy; convert raw fast5 data to fastq format (sequence + quality score)
2	Data Quality Control (QC)	NanoFilt v2.7.1: filter reads with average quality < 10, length < 1400 bp or > 1600 bp; Uchime algorithm (Usearch 10.0.240) + Gold database: remove chimeric sequences to obtain clean reads
3	Taxonomic Annotation & Phylogenetic Tree Construction	Kraken2: taxonomic annotation and OTU table construction; select the highest-quality sequence per species as representative sequence; Muscle + FastTree: phylogenetic tree construction; rarefaction to the minimum

Step	Analysis Item	Core Software/Database & Key Parameters
		sequencing depth for sample normalization
4	Microbial Community Composition Analysis	R 4.0.3 (ggplot2 package): data transformation and visualization of community composition at different taxonomic levels
5	Alpha Diversity Analysis	R 4.0.3: PD index (Picante package), other indices (Vegan package); statistical tests (Wilcoxon rank-sum test, Kruskal-Wallis test via stats package; multiple comparisons via agricolae package)
6	Beta Diversity Analysis	R 4.0.3: Unifrac distance (GuniFrac package), Bray-Curtis/Jaccard distance (vegdits, Vegan package); PCoA (ape package), PCA/NMDS (Vegan package); cluster analysis (hclust, stats package); Anosim/adonis (Vegan package)
7	Differential Species Analysis	LEfSe tool; random forest (randomForest package, R 4.0.3); Metastas analysis (custom R script)
8	Microbial Community Functional Prediction	SILVA 138 database: 16S rRNA gene clustering and annotation; KEGG database: linear transformation of results (corrected by 16S rRNA gene copy number from NCBI) for functional profile prediction
9	Physicochemical Data Analysis	R 4.0.3: Spearman rank correlation (physicochemical factors-species/physicochemical factors correlation); RDA/dbRDA/CCA (rda/capscale/cca, Vegan package); linear regression (alpha diversity-physicochemical factors, stats package); Mantel test (beta diversity-physicochemical factors, Vegan package); visualization of all results in R
